# Effect of Esketamine Pretreatment on Necroptosis in Mice with Trigeminal Neuralgia and Anxiety-Like Behavior via the RIP1/RIP3/MLKL Pathway

**DOI:** 10.1007/s10571-026-01782-y

**Published:** 2026-07-28

**Authors:** Rui Dong, Yuan Tian, Chendi Zhao, Jiaxin Liu, Xin Liu, Zhao Li, Xiaoxue Dong, Shaojun Zhang, Xiuli Wang

**Affiliations:** 1 Department of Pain Medcine, The First Hospital of Qinhuangdao, No. 258 Wenhua Road, Haigang District, Qinhuangdao City, 066000 China; 2 Department of Anesthesiology, Tangshan Workers’ Hospital, Tangshan, 063099 China; 3 Department of Anesthesiology, Affiliated Hospital of Hebei University, Baoding, 071000 China; 4 Department of Anesthesiology, The Third Hospital of Hebei Medical University, 050051 Shijiazhuang, China

**Keywords:** Trigeminal neuralgia, Anxiety-like behavior, Esketamine pretreatment, Necroptosis, RIP1/RIP3/MLKL pathway

## Abstract

To explore the effect of esketamine (ES) pretreatment on necroptosis in mice with trigeminal neuralgia accompanied by anxiety-like behavior and its correlation with the RIP1/RIP3/MLKL signaling pathway. Thirty male C57BL/6J mice were randomized into five groups (*n*=6): Control, Sham, model group (PIONT), PIONT+NS, and PIONT+ES. Trigeminal neuralgia model was established by left infraorbital nerve transection. ES (10 mg/kg, i.p.) was given daily for 5 days preoperatively (preoperative pretreatment) in the PIONT+ES group before molding, and equal-volume saline was administered identically in the PIONT+NS group. Pain thresholds were measured on postoperative days 3, 7, 14 and 21. Behavioral assessments were conducted on day 19, and tissue samples were harvested on day 21. Histopathological staining, immunofluorescence, qPCR, Western blot, ELISA and molecular docking were further performed for subsequent analyses. PIONT group showed reduced pain thresholds, anxiety-like behaviors, neuronal degeneration, decreased dendritic spine density, elevated TNF-α, and upregulated RIP1/RIP3/MLKL (all *P*<0.05). ES pretreatment prevented the development of these pathological alterations, with improved neuropathology, reduced TNF-α/RIP1/RIP3/MLKL levels, and increased spine density (*P*<0.05). Molecular docking showed ES binds RIP1/RIP3/MLKL/TNF-α with energies -5.46, -5.10, -5.94, -7.36 kcal/mol. ES pretreatment prevents the development of trigeminal neuralgia and anxiety-like behaviors in mice, which is associated with suppression of necroptosis-related signaling.

**Graphical Abstract:**

**Figure:**
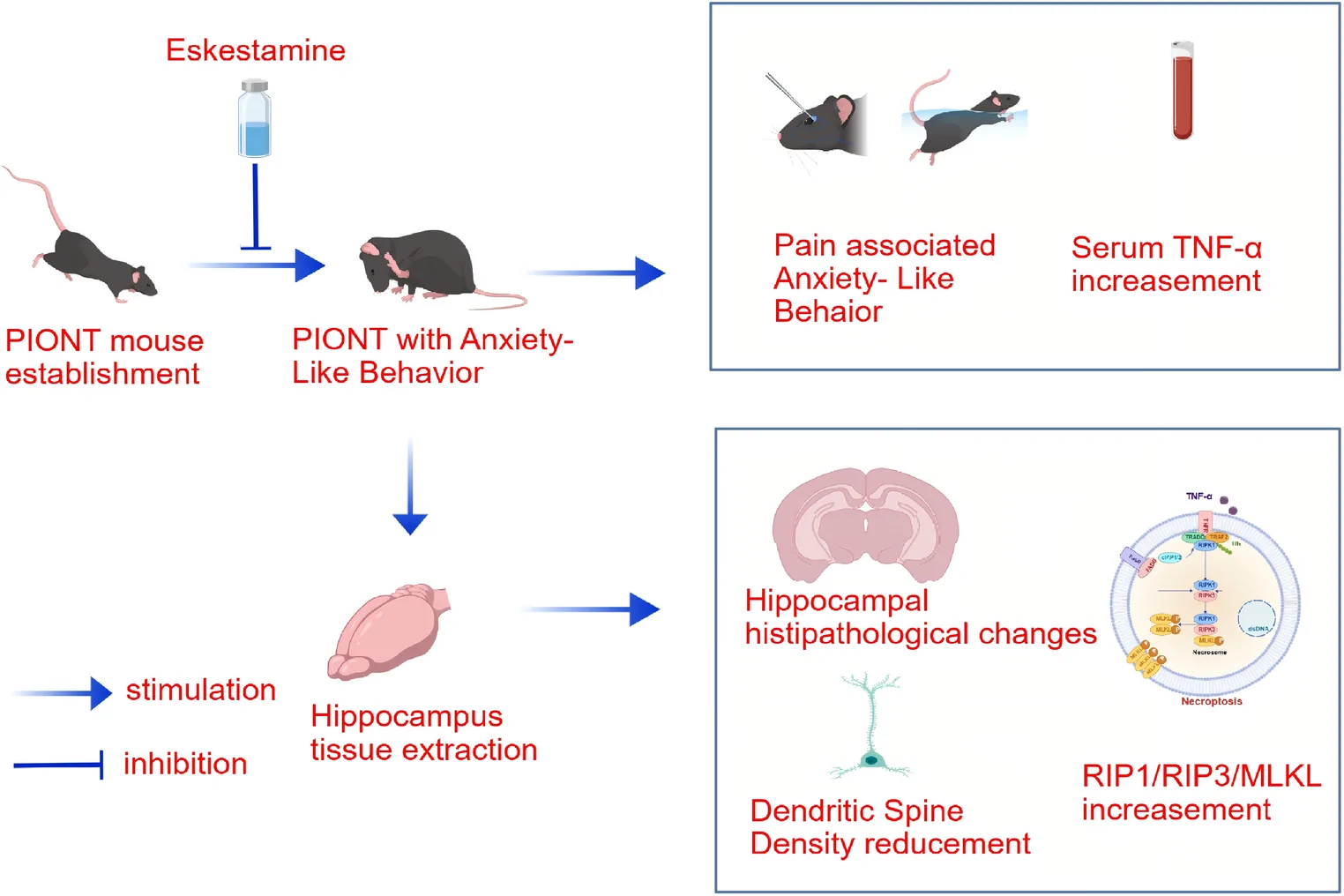

**Supplementary Information:**

The online version contains supplementary material available at https://doi.org/10.1007/s10571-026-01782-y.

## Introduction

Trigeminal neuralgia (TN) is a chronic neuropathic pain disorder with sudden, intense facial pain, severely compromising patients’ quality of life (Jiang et al. 2025; Martinelli et al. 2025). TN patients often present anxiety-like behaviors (comorbidity rate >45%) (Baghaei et al. 2023), forming a bidirectional loop: pain exacerbates emotional distress, while anxiety amplifies pain via central sensitization, increasing treatment complexity. TN-associated anxiety mechanisms remain unclear. Neuroanatomically, TN involves trigeminal ganglion (TG) brainstem trigeminal nucleus thalamocortical pathway activation, retrogradely propagating to the hippocampus (pain-emotion integration center) (Sun et al. 2026). Chronic pain induces hippocampal neuroinflammation and oxidative stress, causing dentate gyrus (DG) neuronal damage and synaptic loss, critical for anxiety-like behaviors (Zhang 2023). The specific molecular link between TN and hippocampal pathology is unclear. Among regulated cell death, necroptosis depends on RIP1/RIP3/MLKL signaling and pro-inflammatory cytokine release (Chen et al. 2024). Unlike apoptosis (caspase-dependent, non-inflammatory) or pyroptosis (inflammasome-mediated, infection-related), necroptosis mediates “neuronal injury-inflammation-pain” cycle in neuropathic pain (Duan et al. 2022; Wang et al. 2025a, b). RIP3 knockout mice show reduced TG neuronal necrosis and elevated pain thresholds, validating necroptosis as a therapeutic target (Wang et al. 2023).

Esketamine (ES), an NMDA receptor antagonist, has rapid antidepressant and analgesic properties. Beyond NMDA antagonism, ES inhibits TNF-α release (blocking RIP1 activation), downregulates MLKL phosphorylation (preventing membrane perforation), and promotes hippocampal synaptic repair via mTOR pathway (Vasiliu 2023; Wang et al. 2023). However, ES’s preventive effects on trigeminal neuralgia with anxiety-like behavior and its role in regulating hippocampal necroptosis via RIP1/RIP3/MLKL pathway remain unclear, limiting targeted preventive strategy development for TN comorbid with emotional disorders (Wang et al. 2025a, b).

The present study postulates that ES pretreatment exerts preventive effects against the development of trigeminal neuralgia and anxiety-like behaviors, which is associated with the suppression of necroptosis-related RIP1/RIP3/MLKL signaling. We aim to elucidate the neuroprotective mechanisms mediated by ES pretreatment and provide experimental evidence for the clinical preventive intervention of trigeminal neuralgia complicated with anxiety: (1) Evaluate ES pretreatment effects on pain thresholds and anxiety-like behaviors; (2) Examine hippocampal necroptosis markers; (3) Analyze the molecular binding association between ES and RIP1/RIP3/MLKL via molecular docking prediction. Findings will provide new insights into preventing the onset of this comorbid condition.

## Materials and methods

### Experimental Animals and Grouping

Male C57BL/6J mice (6–8 weeks old, SPF grade) were housed five per cage under a 12-hour light/dark cycle in a controlled environment with a temperature of 22–24 °C, relative humidity of 50%–70%, and ambient noise below 60 dB. The animal room was well ventilated, and all mice had free access to nutritionally balanced chow and clean drinking water. All mice were purchased from SPF (Beijing) Biotechnology Co., Ltd. (License No.: SCXK(Beijing)2024-0001). Experimental procedures were performed strictly in accordance with the pain research guidelines established by the International Association for the Study of Pain (IASP) (Raja et al. 2020) and were approved by the Animal Ethics Committee of Qinhuangdao First Hospital (No. 2025k-170-01DW). To verify whether ES pretreatment exerts its preventive effects via inhibiting the necroptosis pathway, after one week of adaptive feeding, the mice were randomly divided into five groups (*n* = 6 per group)using a random number table: normal control group (Control), sham operation group (Sham), model group (PIONT), model + normal saline group (PIONT + NS), and model + ES group (PIONT + ES). According to Zhang’s literature, PIONT stands for “partial infraorbital nerve transection” (Zhang 2023). All investigators involved in experimental harvesting and proteomics were blinded to the group allocation throughout the study. Furthermore, the investigators responsible for data processing and statistical analyses remained blinded to group identities until all analyses were completed to ensure an unbiased interpretation of results.

### Main Reagents and Instruments

ES hydrochloride injection (2 ml: 50 mg; National Drug Approval No. H20193336) was purchased from Jiangsu Hengrui Pharmaceutical Co., Ltd. HE staining kit was supplied by Wuhan Elabscience Biotechnology Co., Ltd. (E-IR-R117), and the Golgi staining kit by Guangzhou Weijia Technology Co., Ltd. (CK0059). The mouse TNF-α ELISA kit was purchased from Shanghai Meiyan Biotechnology Co., Ltd. (Ek-M21159). Protein concentration was determined and adjusted using the BCA Protein Quantification Kit (PA115, TIANGEN BIOTECH (BEIJING) CO., LTD., China).The Von Frey Hairs tactile sensitivity test kit was provided by Ugo Basile Life Science (Shanghai) Co., Ltd. (37450-275).The main equipment includes an upright white light microscope (Nikon Eclipse C1, Japan), microscope imaging software (NIS_F_Ver43000_64bit_E, Nikon, Japan), and a microscope imaging system (Nikon digital sight DS-FI2, Nikon, Japan). Additionally, there is an upright fluorescence microscope (Nikon Eclipse C1, Nikon, Japan), fluorescence image synthesis software (AIpathwell, Servicebio), a vibrating microtome (VT1000S, Leica), a tissue section digital scanner (LG-S80, Servicebio), and digital slide viewer software (Saiviewer, Servicebio).

### Model Establishment and Drug Intervention

Mice in the Control group were kept undisturbed in their home cages (Xu et al. 2022), while the remaining four groups were placed in an induction chamber and anesthetized by intraperitoneal injection of 1% sodium pentobarbital (50 mg/kg). After anesthesia, the mice were positioned supine with all limbs fixed to the surgical table. The left cheek skin was disinfected with povidone-iodine. Using ophthalmic scissors, an approximately 5 mm incision was made between 3 mm posterior to the nose and 5 mm below the eye canthus. Under a surgical microscope, the infraorbital nerve, located at the infraorbital foramen of the maxilla, was identified. In the Sham group, only the infraorbital nerve was exposed without transection. In the PIONT, PIONT + NS, and PIONT + ES groups, approximately a 1 mm segment of the nerve within the visual field was partially transected using microsurgical scissors to prevent regeneration and repair, without complete severing. The incision was then sutured, and mice were returned to standard housing conditions for recovery. Mice in the PIONT+ES group received intraperitoneal administration of 10 mg/kg ES once daily for 5 consecutive days before model establishment, while mice in the PIONT+NS group were treated with an equal volume of normal saline.

### Preparation of Tissue Sections

(1) TG:

On day 21 after modeling, mice were anesthetized with 1% sodium pentobarbital, and their limbs were fixed on the surgical table in a prone position. The fur on the head was shaved, and the skin was disinfected with povidone-iodine solution. The skin was incised, and the underlying muscles were carefully separated to expose the skull. At the base of the skull near the zygomatic bone, a cranial drill was used to open a small window in the skull. The surrounding connective tissue of the trigeminal ganglion was gently separated using a microsurgical dissector to expose the trigeminal ganglion. The ganglion was carefully lifted with forceps, excised with surgical scissors, and immediately immersed in 4% paraformaldehyde for fixation overnight at 4 °C. After fixation, the samples were rinsed with PBS and dehydrated sequentially in 70%, 80%, 95%, and 100% ethanol for 30 min at each concentration. The dehydrated tissue was then cleared in xylene for 1 h, embedded in paraffin, and serially sectioned at a thickness of 5–10 μm using a microtome.

(2) Hippocampus:

Anesthesia and pretreatment were identical to those described above. After skull exposure, the hippocampus (an arc-shaped structure beneath the lateral ventricle) was dissected by gently separating surrounding brain tissue with a microsurgical dissector, lifting with forceps, and excising with scissors. Subsequent paraffin embedding and sectioning were the same as for the trigeminal ganglion.

### Methods

#### Measurement of Facial Mechanical Pain Threshold

Facial pain thresholds were assessed at baseline (pre-anesthesia) and postoperative days 3, 7, 14, and 21 using the Von Frey test protocol. Mice were first placed in an elevated wire-mesh testing chamber for 1 h, then transferred to a wide-grid metal platform. A 0.008 g Von Frey filament was used to stimulate the infraorbital nerve distribution area of the face. The filament was gently pressed perpendicularly onto the skin until it bent slightly, maintaining contact for 1–2 s. The force was gradually increased until an obvious pain-related behavior, such as head withdrawal or facial scratching was observed. The stimulus force at this point was recorded as the facial pain threshold. Each test was repeated five times with at least a 2-min interval between stimulations, and the average value was taken.

#### Behavioral Tests

##### Open-Field Test

The open-field test was conducted on day 19 after modeling. Mice were acclimated to the open-field box (40 cm × 40 cm × 30 cm) one day before testing. Tests were carried out between 8:00 a.m. and 11:00 a.m. Each mouse was gently placed in the center of the box facing the same direction, and allowed to explore freely for 15 min while being recorded by a video camera. The total time spent in the central zone was analyzed. The apparatus was cleaned with 75% ethanol between tests to remove odor cues from previous animals.

##### Forced-Swim Test

The forced-swim test was performed on day 20 after modeling. A transparent cylindrical tank (diameter 20–30 cm, height 40 cm) was filled with water (depth 15–20 cm) maintained at 25 ± 1 °C so that the mouse’s hind limbs could not touch the bottom. Each mouse was gently placed into the tank for 6 min. The total immobility time during the last 2 min was recorded, with immobility defined as minimal movements required to keep the head above water. After the test, mice were dried with a towel and placed in a warm environment. The tank was cleaned with 75% ethanol between trials.

#### Hematoxylin-Eosin (HE) Staining

Paraffin sections of the trigeminal ganglion and hippocampus were baked at 60 °C for 2–4 h, then dewaxed in xylene and rehydrated through 100%, 95%, 80%, and 70% ethanol. Sections were stained with hematoxylin for 5–10 min, rinsed with tap water, and differentiated in 1% hydrochloric alcohol until nuclei appeared light blue under the microscope. After washing, sections were counterstained with eosin for 1–3 min, rinsed, dehydrated through graded ethanol, cleared with xylene, and mounted with neutral resin. The morphology, arrangement, and number of neurons, as well as inflammatory cell infiltration in the trigeminal ganglion and hippocampal dentate gyrus (DG) region, were observed under a light microscope (Nikon Eclipse C1).The microscope drawbar was adjusted to the simultaneous observation and imaging mode, and an appropriate magnification objective lens (10×, 20×, 40×) was selected. An appropriate exposure time was set using the computer microscope imaging software to ensure a white image background and bright tissue colors without overexposure. The brightness of the microscope light source and exposure time were maintained constant throughout the entire shooting process. Images were captured using the NIKON digital sight DS-FI2, and analyzed using the NIS_F_Ver43000_64bit_E software.

#### Golgi Staining

Prepared hippocampal tissues were placed in staining cylinders and rinsed three times with distilled water for 5 min each. Tissue blocks were completely submerged in the A+B mixture and impregnated in the dark for 2 weeks. The tissue was then transferred to Solution C and immersed in the dark for 72 h. After dehydration, clearing, and mounting, the hippocampal region was first identified under low magnification and then examined under high magnification to locate the DG region precisely.The slide scanner and SWESlideScanner scanning software were activated, brightfield scanning mode was selected, and the slide was loaded into the scanner. The SWESlideScanner scanning software was operated to automatically or manually identify the scanning area, set the scanning parameters to 41 layers with 20 steps, and initiate scanning by clicking “Start Scanning”. The scanner automatically focused to collect and synthesize images. SVS format scanning files were opened and viewed using Saiviewer biological scanning software. Images of any part of the tissue section at any magnification (1-400×) were observed and captured on the computer. Dendritic branches beyond the first branching node (> 50 μm) were selected, and dendritic-spine density per unit dendritic length in DG granule cells was analyzed using Image-J software.

#### Determination of Serum TNF-α by ELISA

On day 21 after modeling, blood samples were collected from anesthetized mice via retro-orbital puncture. The samples were left at room temperature for 2 h and centrifuged at 3000 r/min for 15 min. The supernatant was collected, and serum tumor necrosis factor-α (TNF-α) levels were determined using an ELISA kit according to the manufacturer’s instructions.

#### Double Immunofluorescence for RIP1, RIP3, MLKL, and NeuN Co-labeling in the Hippocampal DG Region

Paraffin-embedded hippocampal sections were dewaxed and rehydrated, then placed in 0.01 M citrate buffer (pH 6.0) and heated in a microwave until boiling for antigen retrieval, maintained for 10 min, and cooled naturally to room temperature. Sections were incubated in PBS containing 5% fetal bovine serum and 0.3% Triton X-100 for 1 h at room temperature to block nonspecific binding. After removing the blocking solution, mixed primary antibodies were applied: mouse anti-NeuN antibody (1:200, 66836- 1-Ig, Proteintech Group, Inc., China) combined with either rabbit anti-RIP3 antibody (1:100, 17563-1-AP, AB_2178659, Proteintech Group, Inc., China) or mouse anti-MLKL antibody (1:100, 66675- 1-Ig, AB_2882029, Proteintech Group, Inc., China). Sections were incubated overnight at 4 °C in a humidified chamber. After washing with PBS (3 × 10 min), mixed secondary antibodies were applied: 594-Goat Anti-Mouse Recombinant Secondary Antibody (1:200, RGAM004, Proteintech Group, Inc., China) for labeling NeuN, 488-Goat Anti-Rabbit Recombinant Secondary Antibody (1:200, RGAR002, Proteintech Group, Inc., China) for labeling RIP3, and 488-Goat Anti-Mouse Recombinant Secondary Antibody (1:200, RGAM002, Proteintech Group, Inc., China) for labeling MLKL, followed by incubation in the dark for 2 h. After rinsing with PBS, an anti-fade mounting medium containing DAPI (abs9234, Absin Bioscience Inc., China) was applied, and the sections were incubated in the dark for 10 min. Excess liquid was blotted off, and images were captured under a fluorescence microscope. Adjust the fluorescence filter to the ultraviolet channel and observe the DAPI fluorescence of the slide. Open the microscope imaging software (Nikon Eclipse C1, Nikon, Japan) on the computer and select an appropriate exposure time to ensure that the image background is black and the fluorescence brightness is appropriate without overexposure. Select the corresponding filter based on the fluorescence channel of the slide. Keep the same field of view, take a fluorescence image of one channel, switch the filter, and start taking photos of the next fluorescence channel. Maintain the same exposure time for the same channel throughout the entire shooting process. Open the fusion software AIpathwell and click on “Fluorescence Synthesis”. Images of any part of the tissue section at any magnification (1-400x) were observed and captured on the computer. All the imaging was acquired at 200× magnifications in five non-overlapping fields per mouse and then averaged by an investigator blinded to the groups. Three mice per group were used each staining, and images were quantified using ImageJ software and normalized with DAPI intensity.

#### Western Blot Analysis of RIP1, RIP3 and MLKL Expression in the Hippocampus

Hippocampal tissues were thawed on ice and lysed in RIPA buffer for 30 min. Supernatants were collected, and protein concentrations were determined using a BCA assay. Equal amounts of protein were mixed with 5× loading buffer and boiled at 100 °C for 5 min. SDS-PAGE gels were prepared according to target protein molecular weights, and electrophoresis was carried out at 80–120 V. Proteins were transferred to PVDF membranes at 300 mA for 1–2 h (wet transfer). Membranes were blocked in 5% skim milk for 1 h at room temperature, washed with TBST (3 × 10 min), and incubated overnight at 4 °C with primary antibodies including anti-RIP3 antibody (1:1000, AF7942, Affinity Biosciences LTD, China), anti-MLKL antibody (1:1000, DF7412, Affinity Biosciences LTD, China), and anti-Beta Actin antibody (1:10000, 66009- 1-Ig, Proteintech Group, Inc., China) to against RIP3, and MLKL. After washing, membranes were incubated with appropriate HRP-conjugated secondary antibodies (1:1000, A0208, Beyotime Biotech Inc, China) for 1 h at room temperature, washed again, and visualized using ECL reagents. Images were acquired with a Bio-Rad GELDOC Go imaging system, and band intensities were quantified using ImageJ software, with Actin as an internal control.

#### Molecular Docking and Visualization of ES with Necroptosis-Related Proteins

Potential binding domains (active pockets) on target proteins were determined using the Grid function in AutoDock Tools 1.5.7. Flexible molecular docking between ES and necroptosis-related proteins was performed using the DOCK function of the same software, and multiple independent docking runs were carried out. The complex with the lowest binding energy was selected for further analysis. The docked protein–ligand complexes were visualized and rendered using PyMOL, adjusting color, transparency, and display mode to generate high-quality images. Binding energies between ES and target proteins were calculated; as binding energies are typically negative, larger absolute values indicate stronger and more stable interactions.

### Statistical Analysis

Statistical analysis was performed using GraphPad Prism 9. (GraphPad Software, USA). Results were expressed as mean ± standard error of the mean (Mean ± SEM). Multiple group differences were analyzed by one-way ANOVA with Tukey’s post hoc test. Longitudinal repeated-measures data were evaluated via two-way repeated-measures ANOVA. A value of *P* < 0.05 was defined as statistically significant.

## Results

### Comparison of Facial Pain Thresholds Among Groups

See Fig. 1.

**Fig. 1:**
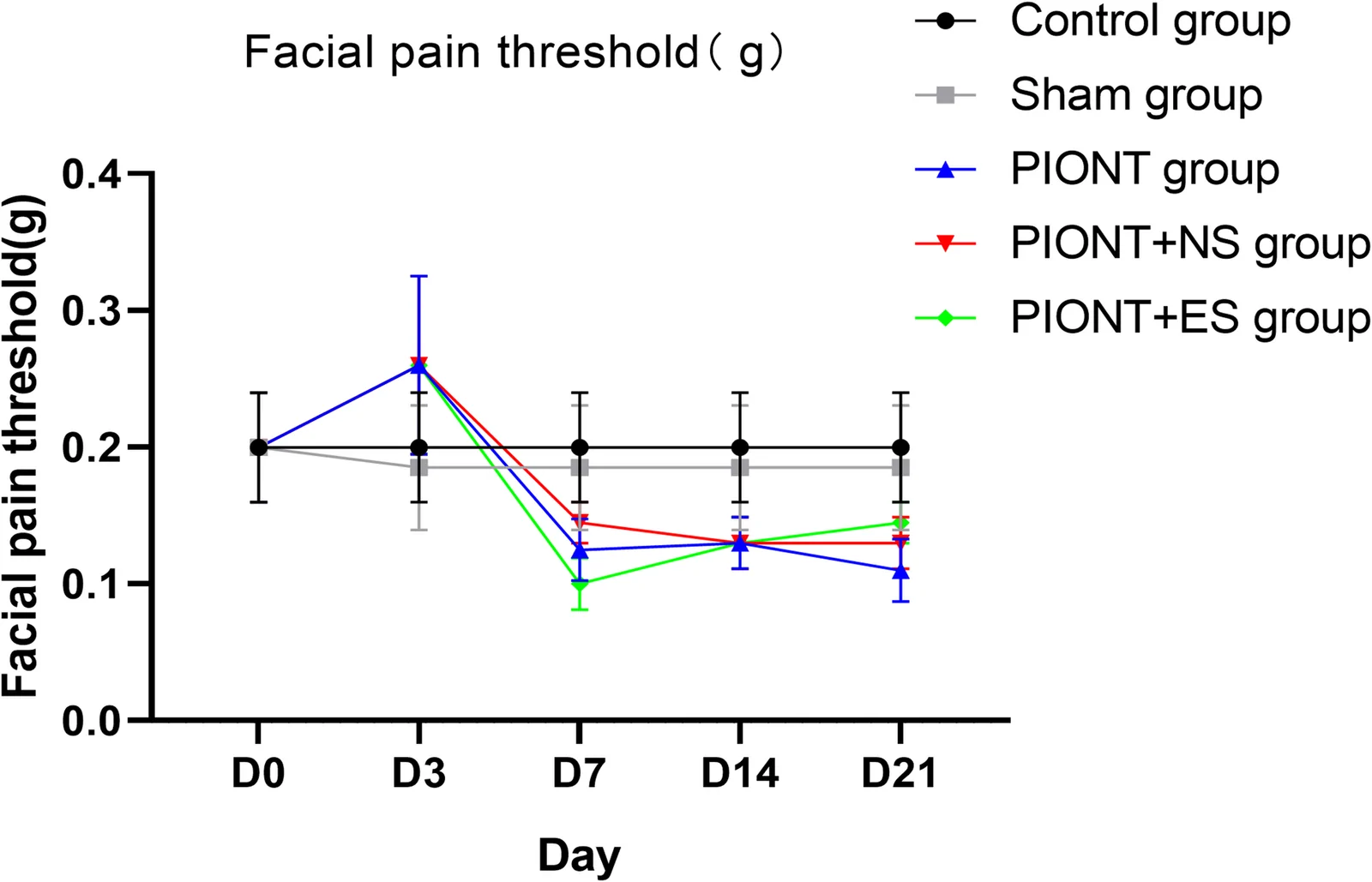
Comparison of facial pain thresholds among groups. Ipsilateral facial pain thresholds were measured at baseline (day 0) and day 3, 7, 14, 21 post-modeling. Data were analyzed by two-way repeated-measures ANOVA (factors: group and time), with post hoc corrections (*n*=6 biological replicates per group). A significant main effect of time was observed (F (4, 125) = 4.318, *P* = 0.0026), demonstrating that facial pain thresholds changed significantly across the time course. In contrast, the main effect of group (F (4, 125) = 0.725, *P* = 0.5762) and the group × time interaction (F (16, 125) = 0.745, *P* = 0.7431) did not reach statistical significance. Compared with the Control and Sham groups, the facial pain threshold of mice in the PIONT group showed no significant change on day 3 post-modeling, but was significantly reduced from day 7 to day 21 post-modeling, with persistent hyperalgesia (*P*<0.05). The PIONT+ES group had slightly higher pain threshold values than PIONT and PIONT+NS groups at days 14 and 21, but this minor numerical trend does not represent statistically meaningful intergroup differences. These data only suggested a trend of prevention against pain hypersensitivity development in the PIONT+ES group, rather than confirming ES significantly relieved established pain hypersensitivity. The evident directional trend may be masked by inter-individual variation among animals; increasing sample size will be adopted in follow-up studies to further verify this tendency

### Comparison of Open-Field Test Results Among Groups

See Fig. 2.

**Fig. 2:**
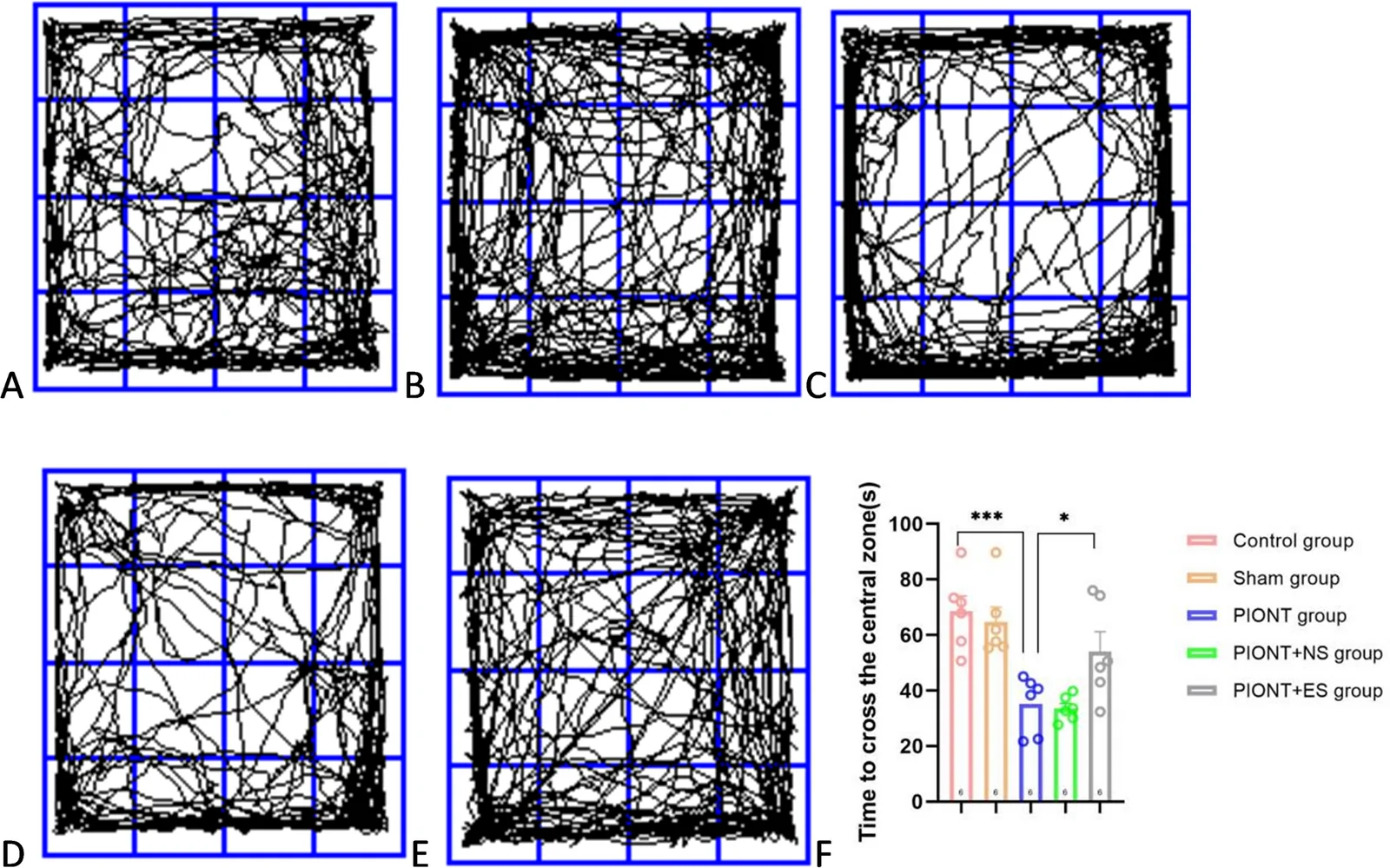
Comparison of open-field test results among groups. **A** through **E** represent the typical graphs of different groups. **A** Control group; **B** Sham group; **C** PIONT group; **D** PIONT + NS group; **E** PIONT + ES group. **F** Statistical analysis was performed using one-way ANOVA (*n*=6 biological repetition per group), *P<0.05, **P<0.01, ***P<0.001, ns: no significant difference, data presented as mean±SEM. On day 19 post-modeling, mice in the PIONT group halved the time spent crossing the central area compared to the Control group (***P<0.001). In contrast, mice in the PIONT + ES group significantly increased time spent crossing the central area by 0.59-fold versus PIONT alone (*P<0.05)

### Comparison of Forced-Swim Test Results Among Groups

See Fig. 3.

**Fig. 3:**
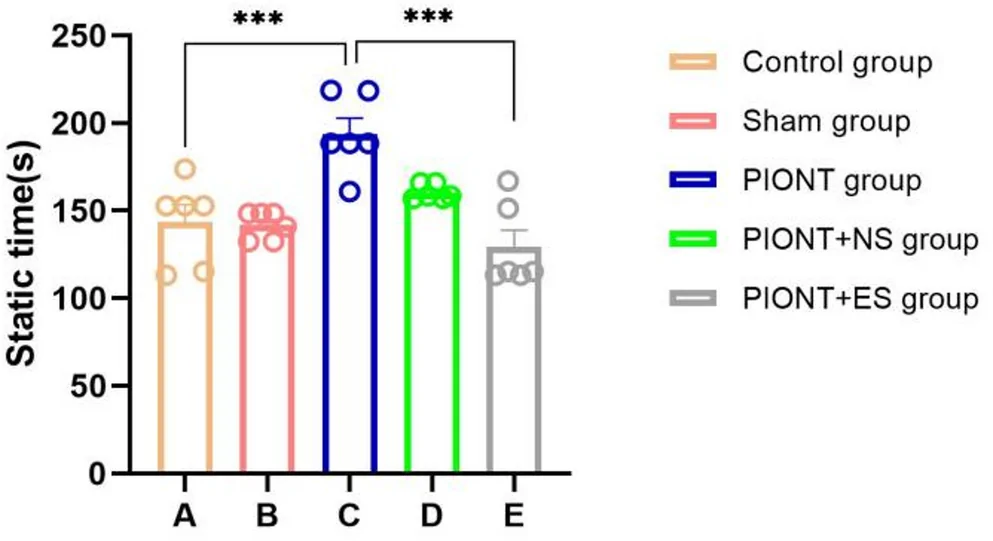
Comparison of forced-swim test results among groups. **A** Control group; **B** Sham group; **C** PIONT group; **D** PIONT + NS group; **E** PIONT + ES group. Statistical analysis was performed using one-way ANOVA (*n*=6 biological repetition per group), *P<0.05, **P<0.01, ***P<0.001, ns: no significant difference, data presented as mean±SEM.On day 19 post-modeling, mice within the PIONT group displayed a markedly prolonged immobility time, surpassing that of the Control group by 1.3-fold (***P<0.001). In stark contrast, mice in the PIONT + ES group showed alleviated depressive-like behaviors mediated by ES preventive pretreatment, with immobility time plummeting by 19% compared to the PIONT group (***P<0.001)

### Histopathological Changes in the Trigeminal Ganglion and Hippocampal DG Region in Each Group

**Fig. 4:**
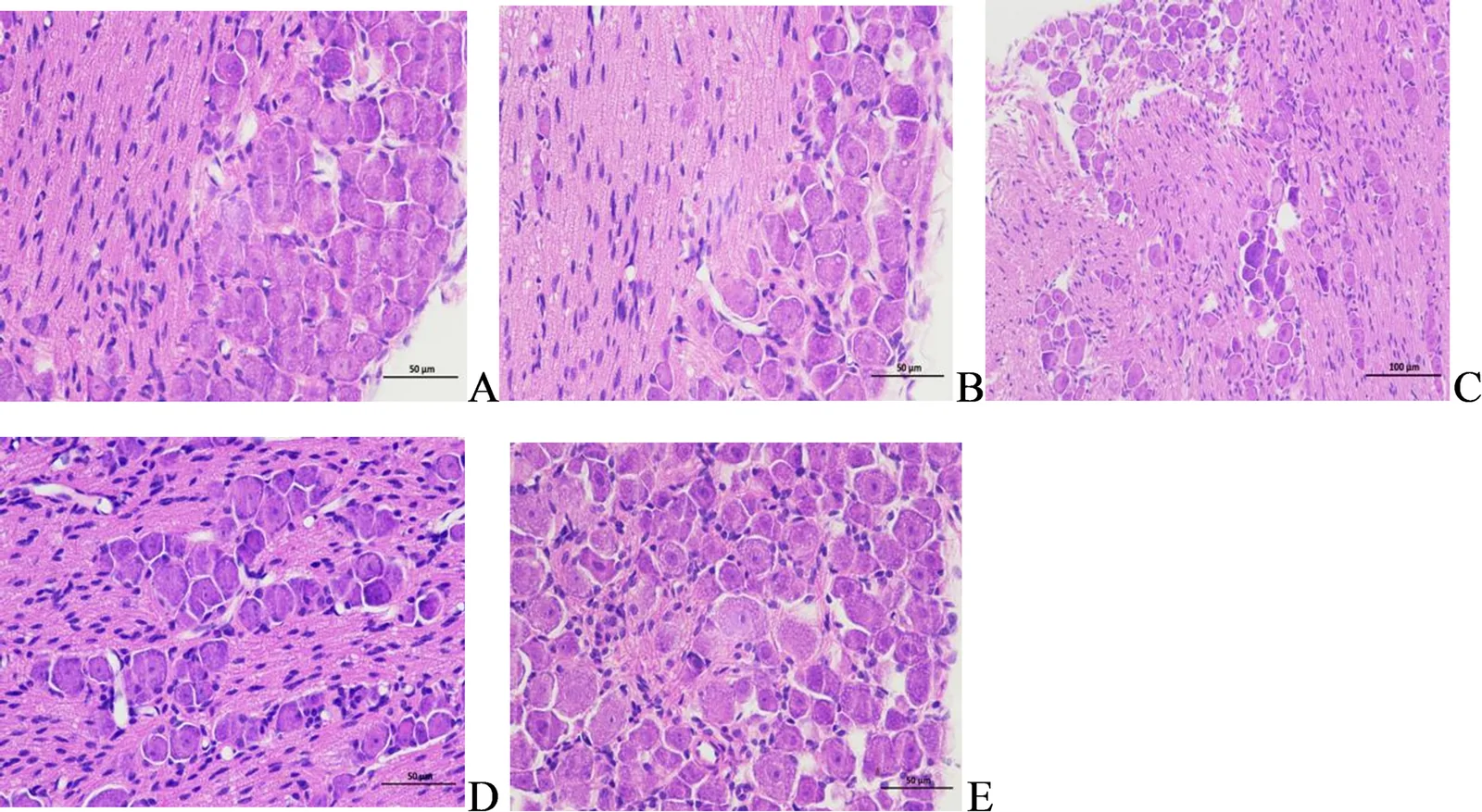
Pathological morphological changes of trigeminal ganglion neurons in each group (HE staining, ×400). **A** through **E** represent the typical graphs of different groups. **A** Control group; **B** Sham group; **C** PIONT group; **D** PIONT + NS group; **E** PIONT + ES group (*n* = 3 biological repetition per group). In the Control and Sham groups, the trigeminal ganglion neurons were neatly arranged with normal morphology, no obvious degeneration or necrosis, abundant cytoplasm, and clear nucleoli (**A**, **B**). In the PIONT group, trigeminal ganglion neurons showed degeneration and necrosis, disorganized arrangement, cytoplasmic condensation in some cells, and nuclear pyknosis or disappearance (**C**). The PIONT + NS group exhibited pathological changes in the trigeminal ganglion similar to those in the PIONT group (**D**). In the PIONT + ES group, the degree of degeneration and necrosis of trigeminal ganglion neurons was milder than that in the PIONT + NS group. Neuronal arrangement appeared more orderly, and some cells showed nearly normal morphology (**E**)

**Fig. 5:**
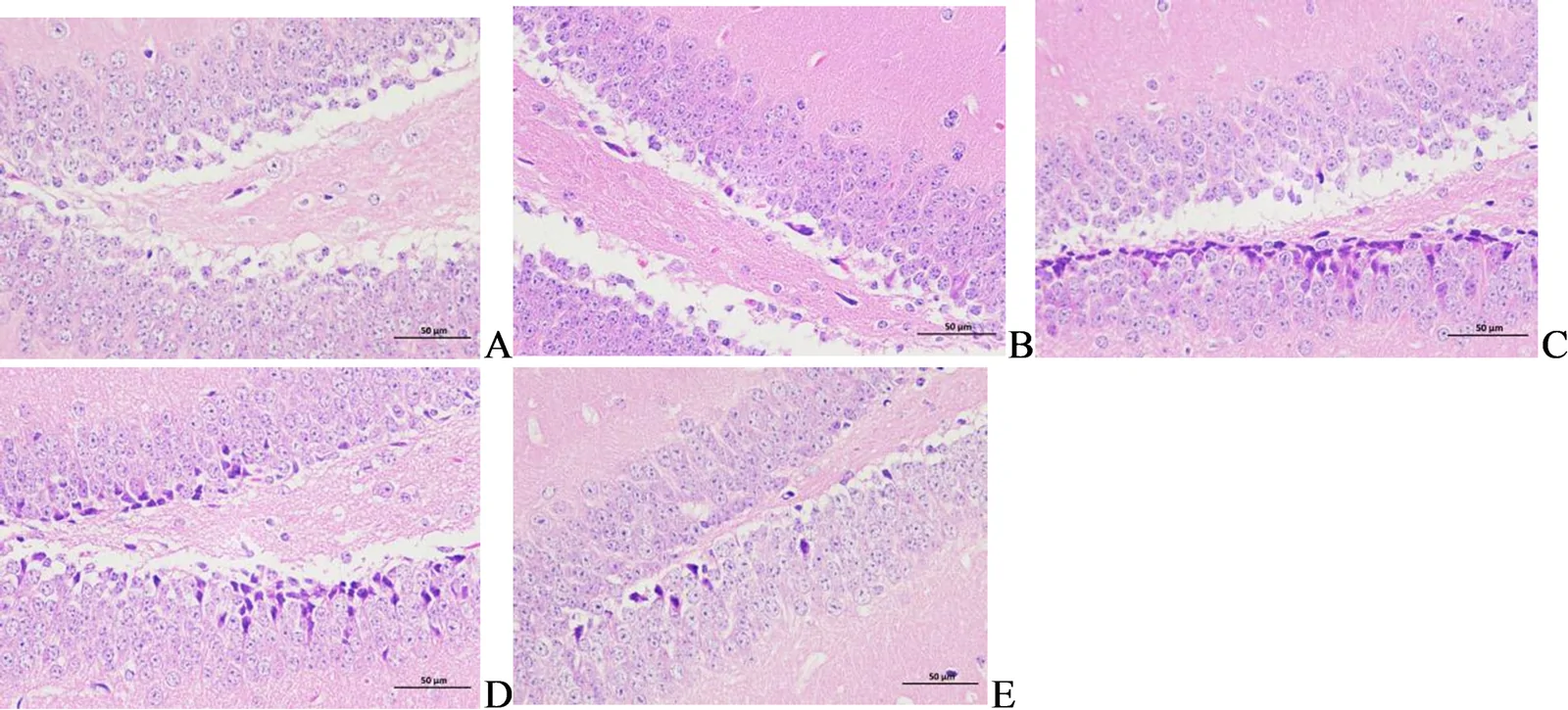
Pathological morphological changes of neurons in the hippocampal DG region in each group (HE staining, ×400). **A** Control group; **B** Sham group; **C** PIONT group; **D** PIONT + NS group; **E** PIONT + ES group (*n*=3 biological repetition per group). In the hippocampal DG region, neurons were densely arranged, with intact morphology, uniform cytoplasm, and distinct nucleoli, showing no apparent pathological changes (**A**, **B**). The hippocampal DG region displayed a reduced number of neurons, abnormal cell morphology, uneven cytoplasm, and indistinct nucleoli (**C**). The PIONT + NS group exhibited pathological changes inhippocampal DG region similar to those in the PIONT group (**D**). As a preventive effect of ES pretreatment, the PIONT + ES group showed increased neuronal number, improved cell morphology, and more prominent nucleoli in the hippocampal DG region compared with the PIONT + NS group (**E**)

On the 21st day after modeling, anxiety-like behaviors in mice were stable; therefore, this time point was regarded as the successful establishment of the trigeminal neuralgia model with anxiety-like behavior. The trigeminal ganglion and hippocampal DG regions were collected on day 21 and subjected to HE staining (Figs. 4 and 5).

### Comparison of Dendritic Spine Density per Unit Dendritic Length of Granule Cells in the Hippocampal DG Region Among Groups

See Fig. 6.

**Fig. 6:**
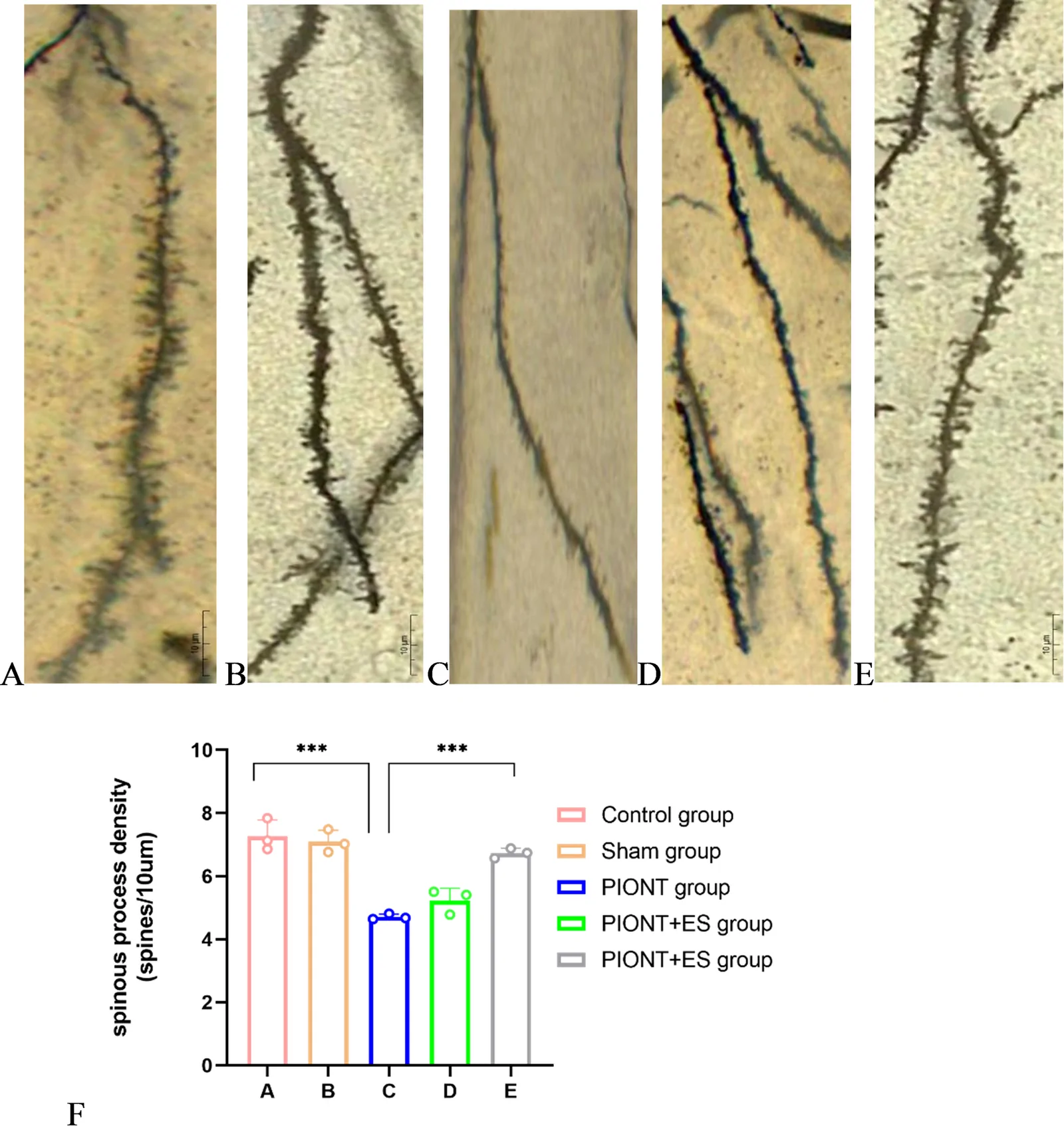
Comparison of dendritic spine density per unit dendritic length of granule cells in the hippocampal DG region among groups (Golgi staining, X5). **A** through **E** represent the typical graphs of different groups. **A** Control group; **B** Sham group; **C** PIONT group; **D** PIONT + NS group; **E** PIONT + ES group, **F** Comparison of dendritic spine density per unit dendritic length of granule cells in the hippocampal DG region among groups (*n* = 3 biological replicates per group; mean value from six randomly selected fields per replicate). In the Control and Sham groups, granule cells within the hippocampal DG region exhibited high dendritic spine density per unit dendritic length, with spines evenly distributed and displaying regular morphology (**A**, **B**). Conversely, the PIONT group revealed a significant reduction in dendritic spine density within the hippocampal DG region; spines were fewer, unevenly distributed, and some exhibited abnormal morphology (**C**). The PIONT + NS group mirrored the PIONT group, maintaining a significantly lower spine density than the Control and Sham groups, with no discernible improvement in spine number or distribution (**D**). In stark contrast, the PIONT + ES group demonstrated a substantial increase in dendritic spine density compared to the PIONT group, approximating levels seen in the Control and Sham groups. Spines were more uniformly distributed and displayed nearly normal morphology (**E**). Statistical analysis employed one-way ANOVA (*n* = 3 biological replicates per group; mean value from six randomly selected fields per replicate; *P<0.05, **P<0.01, ***P<0.001; ns: no significant difference; data presented as mean±SEM). Quantitative analysis confirmed that, compared to the control group, the PIONT group exhibited a significant 0.53-fold reduction in dendritic spine density within the hippocampal dentate gyrus (DG) region on day 21 post-modeling (*n* = 3 biological replicates per group; mean value from six randomly selected fields per replicate; ***P<0.05). Furthermore, compared with the PIONT group, the PIONT + ES group showed a marked increase in dendritic spine density, reaching 1.4-fold higher levels on day 21 (*n* = 3 biological replicates per group; mean value from six randomly selected fields per replicate, ***P < 0.001) (**F**)

### Comparison of Serum TNF-α Levels Among Groups

See Fig. 7.

**Fig. 7:**
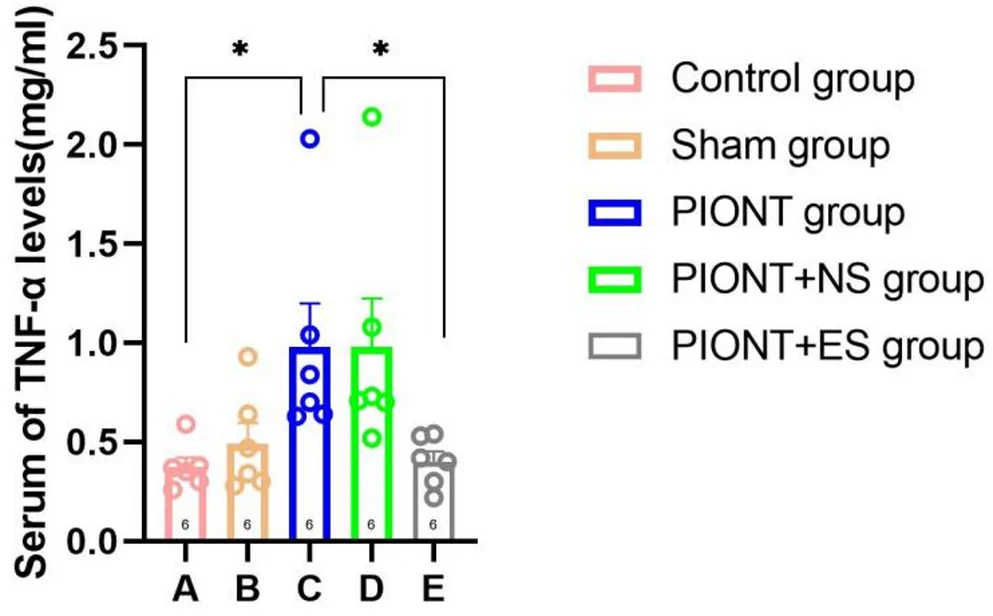
Comparison of serum TNF-α levels among groups. Statistical analysis was performed using one-way ANOVA (*n*=6 biological repetition per group), *P<0.05, **P<0.01, ***P<0.001, ns: no significant difference, data presented as mean±SEM. Compared with the Control group, mice in the PIONT group exhibited a marked 2.6-fold increase in serum TNF-α levels (*P < 0.05). In contrast, the PIONT + ES group demonstrated a markedly greater reduction of 0.59-fold in serum TNF-α levels compared to the PIONT group (#*P* < 0.05)

### Comparison of the Number of Neurons Co-labeled with RIP3 and MLKL and NeuN in the Hippocampal DG Region Among Groups

**Fig. 8:**
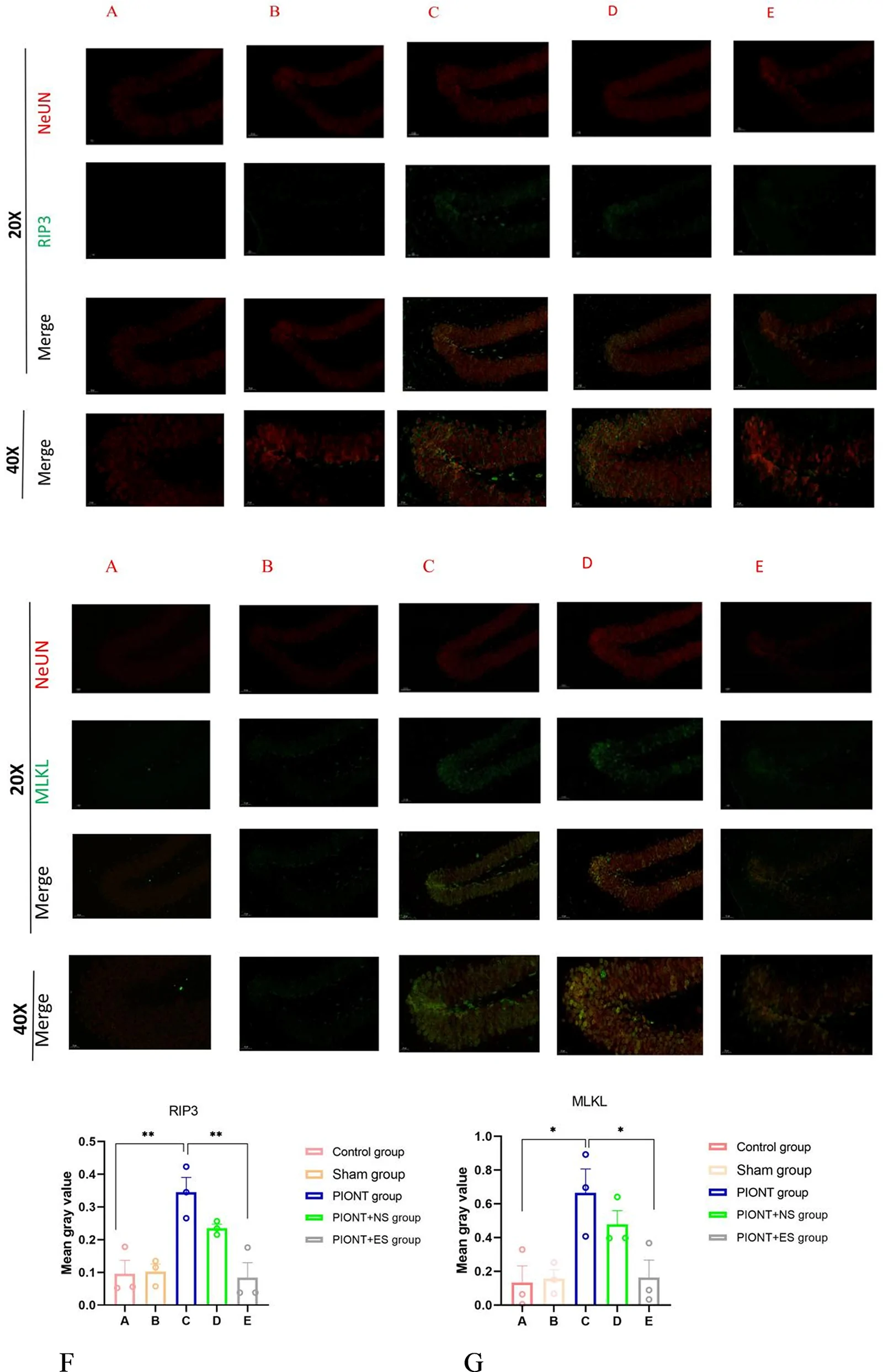
Immunofluorescence staining results. **A** Control group; **B** Sham group; **C** PIONT group; **D** PIONT + NS group; **E** PIONT + ES group (*n*=3 biological repetition per group). **A** through **E** represent the typical graphs of different groups. Double immunofluorescence staining revealed that in both the Control and Sham groups, neurons within the hippocampal DG region displayed normal morphology, featuring a substantial population of NeuN-positive neurons. The fluorescence signals for RIP3 and MLKL remained faint, and the count of neurons co-labeled with NeuN was low (**A**, **B**). Conversely, the PIONT group exhibited a marked reduction in neuronal numbers within the hippocampal DG region, with some neurons displaying abnormal morphology. The fluorescence intensity of RIP3 and MLKL intensified significantly, accompanied by a substantial increase in NeuN co-labeled neurons (**C**). Results for the PIONT + NS group closely mirrored those of the PIONT group (**D**). In the PIONT + ES group, however, the number of neurons in the hippocampal DG region rebounded, their morphology appeared largely restored, and the RIP3 and MLKL fluorescence signals diminished markedly. Critically, the number of NeuN co-labeled neurons significantly decreased compared to both the PIONT and PIONT + NS groups (**E**)

Statistical analysis, performed using one-way ANOVA (*P* < 0.05; data presented as mean±SEM), compared the mean grayscale values of RIP3 and MLKL across groups (Fig. 8F and G). Relative to the Control group, the PIONT group demonstrated a dramatic surge in RIP3 and MLKL protein expression levels within the hippocampal region, escalating by 3.6-fold and 5-fold, respectively (**P < 0.01, *P < 0.05). In contrast, the PIONT + ES group exhibited significantly suppressed expression levels of RIP3 and MLKL proteins in the hippocampus compared to the PIONT group, decreasing by 0.76-fold and 0.75-fold (**P < 0.01 , *P < 0.05).

### Comparison of RIP1, RIP3, and MLKL Protein Expression Levels in the Hippocampal Region Among Groups

See Fig. 9.

**Fig. 9:**
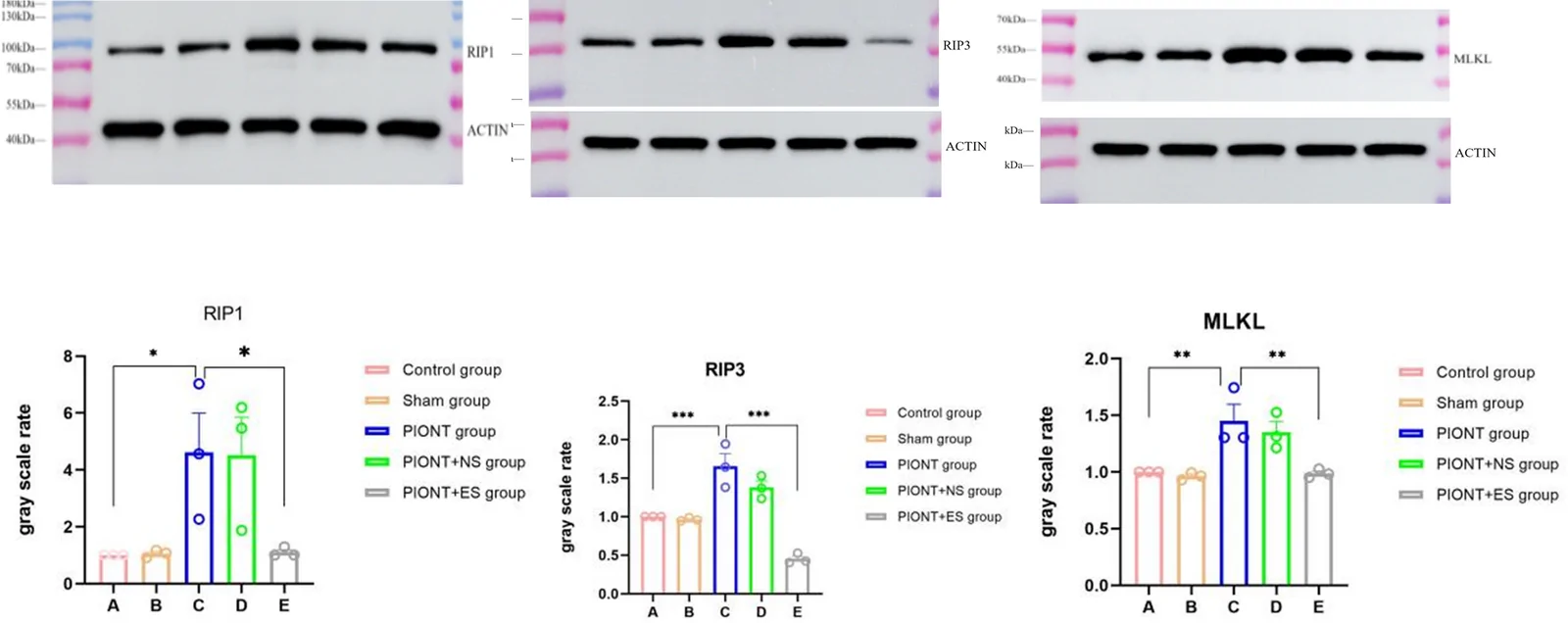
Western blot results. **A** Control group; **B** Sham group; **C** PIONT group; **D** PIONT + NS group; **E** PIONT + ES group. Statistical analysis was performed using one-way ANOVA (*n*=3 biological repetition per group), *P<0.05, **P<0.01, ***P<0.001, ns: no significant difference, data presented as mean±SEM. Western blot analysis revealed that, compared to the Control group, the PIONT group demonstrated significantly increased expression levels of RIP1, RIP3, and MLKL proteins in the hippocampus, rising by 4.6-fold, 1.6-fold, and 1.5-fold, respectively (*P<0.05,***P<0.001,**P<0.01). In contrast to the PIONT group, the PIONT + ES group exhibited significantly decreased expression levels of necroptosis-related RIP1, RIP3, and MLKL markers in the hippocampus, reduced by 0.76 fold, 0.73 fold, and 0.32 fold respectively (*P<0.05,***P<0.001,**P<0.01)

### Molecular Docking Analysis and Visualization Results of ES with Necroptosis-Related Proteins

**Fig. 10:**
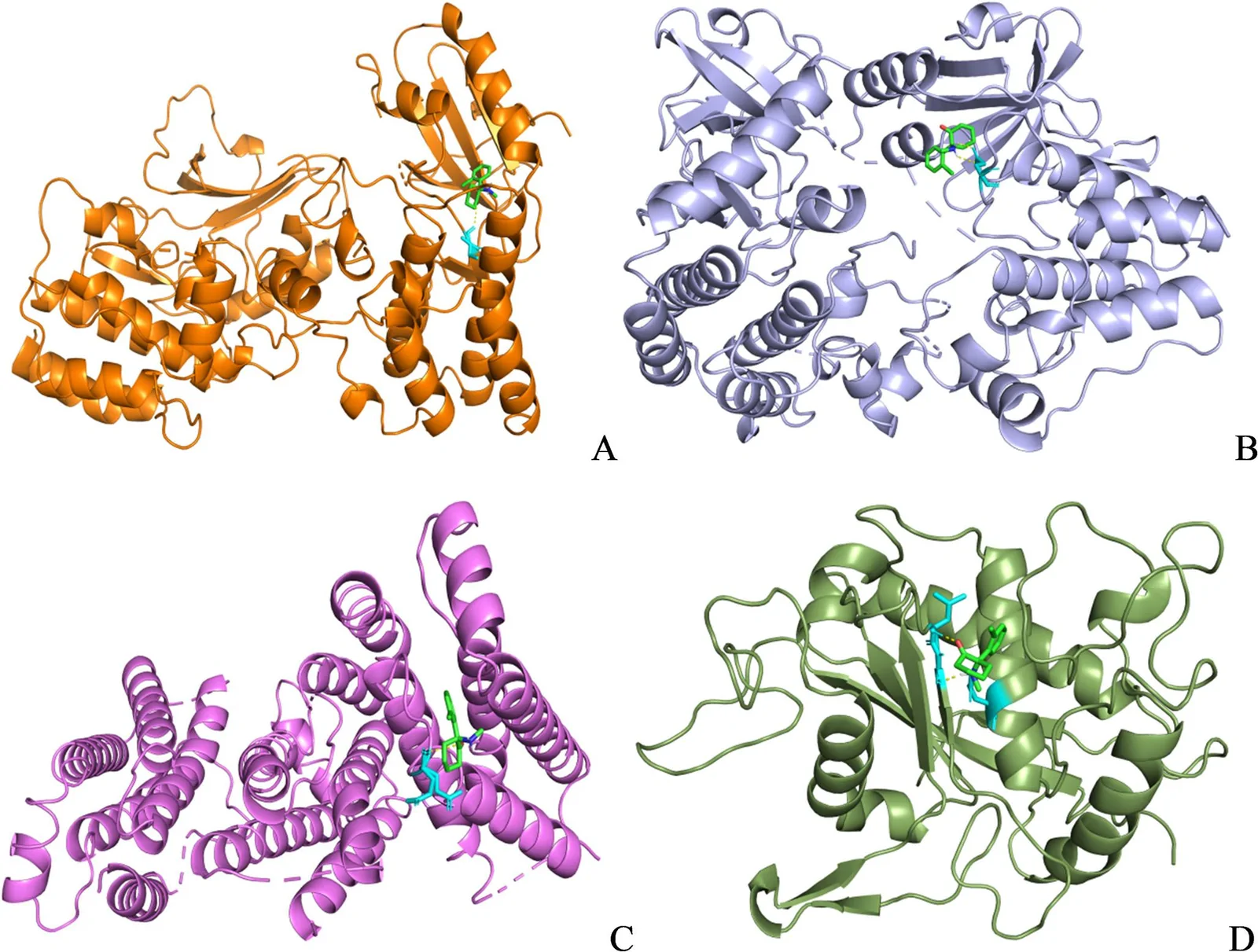
Molecular docking analysis and visualization results of ES with necroptosis-related proteins. **A** ES with RIP1; **B** ES with RIP3; **C** ES with MLKL; **D** ES with TNF-α

The binding energies of ES with RIP1, RIP3, MLKL, and TNF-α were -5.46 kcal/mol, -5.10 kcal/mol, -5.94 kcal/mol, and-7.36 kcal/mol, respectively (Fig. 10).

## Discussion

TN is a common craniofacial pain syndrome with a bidirectional pain-anxiety comorbidity and unmet therapeutic needs, where necroptosis emerges as a key pathological link. TN affects 4.3/100,000 individuals annually with a 1.5:1 female predominance; 68% of patients develop comorbid anxiety/depression, as emotional distress and persistent pain mutually exacerbate each other via central sensitization (Raja et al. 2020; Bauer et al. 2024; Zhang 2023).Current therapies (SNRIs, cognitive-behavioral therapy, neuromodulation) achieve clinical remission in only 32% of patients, highlighting the need for mechanism-based interventions targeting both pain transmission and emotional processing pathways(Bauer et al. 2024).Necroptosis, a caspase-independent programmed cell death pathway, links peripheral nerve injury to central sensitization in neuropathic pain through sequential phosphorylation of RIP1, RIP3, and MLKL, releasing DAMPs that activate glial signaling cascades(Hu et al. 2021; Duan et al. 2022). Transcriptomic studies in trigeminal ganglia show upregulated RIP3/MLKL in rat TN models, with peak phosphorylation correlating temporally with mechanical allodynia(Xu et al. 2022). This aligns with our findings of elevated expression of necroptosis-related markers in hippocampal neurons post-PIONT, indicating conserved pathological mechanisms across rodent and human TN.

This study investigated the modulatory effects of ES pretreatment on the RIP1/RIP3/MLKL necroptosis signaling pathway in a murine model of trigeminal neuralgia with comorbid anxiety-like behaviors. Using the PIONT model(Xu et al. 2022), we integrated behavioral phenotyping, molecular signaling analysis, and pharmacodynamic profiling to test two hypotheses: (1) Hippocampus necroptosis contributes to pain hypersensitivity and anxiety-like behaviors; (2) ES pretreatment exerts preventive effects against symptom onset via downregulation of necroptosis-related signaling markers. Von Frey testing confirmed that PIONT mice developed mechanical allodynia starting at postoperative day 3 and sustained persistent hyperalgesia up to day 21, consistent with clinical presentations of prolonged pain states in patients with TN (Bauer et al. 2024). Two-way repeated-measures ANOVA showed only a significant time main effect, with non-significant group main effect and group×time interaction. Although PIONT+ES group showed numerically higher pain thresholds at late time points, this was merely a trend rather than statistically significant intergroup difference, possibly masked by animal individual variation; larger sample sizes will be used in follow-up experiments for further validation. We hypothesized that this mild trend might imply that ES pretreatment modulates pain transmission and chronic neuroplastic changes, potentially via the inhibition of necroptosis to restrain the progression of neuropathic pain (Huang et al. 2023). ES exerts bidirectional preventive effects against TN-associated pain-emotion comorbidity onset by targeting shared neural substrates linked to necroptosis-mediated neuroinflammation. Open-field test (OFT) and forced-swim test (FST) revealed PIONT mice had 49% reduced central zone exploration (*p*<0.001, anxiety-like behavior) and 1.3-fold increased immobility time (*p*<0.001, depressive-like phenotype), consistent with human TN reports (Zhang 2023). ES pretreatment normalized these behaviors (59% increased central zone exploration, 19% decreased immobility time vs. PIONT+NS group; *p*<0.05/*p*<0.001), with emotional improvement occurring despite partial pain relief—challenging the “pain-first” paradigm. Mechanistically, this suggests ES targets shared neural substrates (amygdala, periaqueductal gray), where necroptosis-mediated neuroinflammation affects both affective processing and pain modulation (Duan et al. 2022; Wang et al. 2024a, b). Behavioral improvements were accompanied by reduced circulating serum TNF-α concentrations, which linking the downregulation of necroptosis and inflammatory markers to improved behavioral outcome (Duan et al. 2022). Notably, we did not quantify local TNF-α protein levels within the trigeminal nucleus caudalis or prefrontal cortex in the current study and future assays targeting regional central TNF-α expression are warranted to clarify site-specific neuroinflammatory alterations.

ES pretreatment exerts neuroprotective preventive effects against TN pathogenesis by reducing hippocampal necroptosis marker expression and preserving synaptic architecture. Histopathological analysis of trigeminal ganglia (TG) and hippocampal tissues revealed ES pretreatment’s neuroprotective mechanisms. PIONT hippocampal sections revealed striking necroptotic hallmarks: a 3.6-fold surge in RIP3-positive neurons and a 5-fold jump in MLKL-positive neurons (*p*<0.05), accompanied by pronounced cytoplasmic vacuolation and perineuronal edema relative to controls. This mirrors RIP3 upregulation documented in human TN autopsy studies.(Xu et al. 2022) ES pretreatment dramatically curtailed RIP3 and MLKL immunoreactivity by three-quarters (*p*<0.01) and preserved intact neuronal architecture. Remarkably, ES pretreatment protected hippocampal dentate gyrus (DG) synapses, triggering a 1.4-fold increase in dendritic spine density (*p*<0.05) compared to PIONT group mice. This aligns with ES pretreatment’s BDNF-dependent spinogenesis (Huang et al. 2023), suggesting dual mechanisms: the reduction of necroptosis-related marker expression alleviates neuronal loss, while synaptic remodeling restores emotional circuitry function.

Analysis of TNF-α/RIP1/RIP3 level suggests the neuroinflammation-necroptosis link. PIONT mice revealed a striking 2.6-fold surge in serum TNF-α. Remarkably, ES pretreatment slashed TNF-α levels by 59% (all *p*<0.05), significantly reduced the expression levels of RIP1 and RIP3 necroptosis markers by 76% and 73%, respectively (*p*<0.05, *P*<0.001), and significantly curtailed MLKL by 32% (*p*<0.01). These findings extend previous work, showing ES pretreatment modulates necroptotic machinery beyond NMDA receptor antagonism (Li et al. 2023). Notably, sexual dimorphism underscores the critical need for gender-stratified pain research, which may explain the female predominance (Bauer et al. 2024)in TN.

Molecular and computational analyses further elucidated ES’s mechanism. PIONT mice showed 4.6-, 1.6-, and 1.5-fold upregulation of RIP1, RIP3, and MLKL protein in hippocampus (*P<0.05, ***P<0.001, **P<0.01). ES pretreatment normalized expressions, reducing proteins by 32%-76% (*p*<0.05). Molecular docking suggested potential binding affinity of ES pretreatment for TNF-α (-7.36 kcal/mol), followed by MLKL (-5.94 kcal/mol), RIP1 (-5.46 kcal/mol), and RIP3 (-5.10 kcal/mol). Importantly, these in silico findings should be interpreted as hypothesis-generating rather than definitive evidence of direct target engagement, as binding energies alone do not establish biologically meaningful interactions between ES pretreatment and RIP1, RIP3, MLKL, or TNF-α. The docking results are consistent with the in vivo observations and provide complementary preliminary insights supporting the hypothesis that ES pretreatment may modulate necroptotic signaling through potential multi-target interactions, conferring a potential advantage over selective RIP1 inhibitors such as necrostatin-1, which are limited by poor blood–brain barrier permeability (Wang et al. 2024a, b). Besides, molecular docking also revealed a potentially high predicted affinity of ES for TNF-α (-7.36 kcal/mol), which again serves as a preliminary, hypothesis-generating observation requiring further experimental verification.

Notably, the observed trend-level mild pain improvement coupled with near-complete emotional normalization strikingly challenges the “pain-first” paradigm, suggesting independent modulation of affective circuits, a critical finding given that 42% TN patients discontinue treatment due to persistent mood symptoms despite pain control (Zhang 2023; Chen et al. 2025). Our findings suggest ES pretreatment acts as a multifaceted preventive strategy for TN onset, which is closely linked to the RIP1/RIP3/MLKL necroptosis pathway. It restrains the development of pain and emotional comorbidities through convergent regulatory mechanisms associated with the downregulation of necroptosis-related marker expression.

## Conclusion

This study supports the potential protective relevance of ES pretreatment and suggests the involvement of necroptosis-related RIP1/RIP3/MLKL signaling in its beneficial effects. By reducing neuronal necroptosis, preserving synaptic architecture, and normalizing emotional behaviors, we provide a mechanistic framework for ES pretreatment’s clinical preventive application in TN. Specifically, ES pretreatment partially prevents the onset of pain and anxiety-like symptoms in TN mice with anxiety-like behaviors, and improves neuropathological features and hippocampal neuronal structure. These effects are associated with suppression of necroptosis-related signaling, reducing serum TNF-α concentrations, and modulating necroptosis-convergent neuroprotective mechanisms. A critical implication is that targeting necroptosis may address unmet needs for disease-modifying TN therapies, shifting the focus from symptom management to neuroprotection. To translate these findings clinically, future research should focus on: (1) sex-specific pharmacodynamic profiling to optimize ES dosing; (2) targeted delivery systems for enhanced trigeminal ganglion penetration; (3) RIP3 inhibitor combination therapy for synergy; and (4) clinical trials exploring perioperative preventive administration in TN patients at high risk of postoperative neuropathic pain and mood comorbidities. These efforts may transform TN treatment to a pathway-directed approach addressing root pathophysiology.

## Limitations

This study has several limitations that constrain its generalizability and interpretive robustness, which should be addressed in future research: (1) Single rodent model and small sample size constrain statistical power and generalizability; (2) No long-term safety/efficacy data, findings limited to short-term preventive outcomes; (3) Translatability of ES dose to humans unclear, only single dose/route examined; (4) Potential nonspecific motor/sedative effects on behavioral tests not fully excluded; (5) Pathway specificity not validated, future studies needed with RIP3 knockout mice and Necrostatin-1 controls; (6) Limited sample sizes for key analyses, particularly immunofluorescence and dendritic spine measurements, compromise the statistical power and interpretive robustness. Despite these constraints, the current study preliminarily demonstrates ES pretreatment’s potential in preventing the emergence of anxiety-like behaviors secondary to neuropathic pain, laying crucial groundwork for further in-depth investigation.

## Supplementary Information

Below is the link to the electronic supplementary material.

Supplementary Material 1

## Data Availability

No datasets were generated or analysed during the current study.
